# Remodeling of Gut Microbiota Metabolic Function Is Associated with the Therapeutic Effects of Wenweishu Capsules in Chronic Gastritis

**DOI:** 10.3390/ijms27156695

**Published:** 2026-07-27

**Authors:** Zhenxin Ren, Lumei Zhang, Hengcai Hu, Zhiwei Wu

**Affiliations:** 1 College of Biological Science and Technology, Yili Normal University, Yining 835000, China; 2 Xinjiang Key Laboratory of Lavender Conservation and Utilization, Yining 835000, China; 3 School of Basic Medical Sciences, Gansu University of Chinese Medicine, Lanzhou 730030, China

**Keywords:** Wenweishu capsules, wistar rats, gut microbiota, chronic gastritis, high-throughput sequencing

## Abstract

Chronic gastritis (CG) is linked to gut–gastric axis dysbiosis. Wenweishu Capsules (WWS) are clinically used to treat CG, but their concurrent regulatory effects on this axis remain unclear. A CG rat model was established using sodium deoxycholate, ammonia, ethanol, and irregular feeding. Rats received WWS (0.38 g·kg^−1^·d^−1^) for 4 weeks. Gastric lesions, inflammation, and gut microbiota were evaluated via histopathology, ELISA, 16S rRNA sequencing, PICRUSt prediction, and correlation analysis. WWS improved general condition, food intake, and weight gain in CG rats, alleviated gastric mucosal damage, and reduced serum TNF-α and IL-6. These effects were associated with altered IκBα expression and NF-κB pathway modulation. WWS reversed gut microbiota dysbiosis, restored α- and β-diversity, increased *Lachnospiraceae* NK4A136 group abundance, and suppressed potential pathogens. Functional prediction showed that WWS upregulated beneficial pathways such as carbohydrate metabolism and downregulated infection-related pathways. Correlation analysis linked TNF-α/IL-6 negatively with the *Lachnospiraceae* NK4A136 group and positively with Bacteroides. WWS ameliorates CG through a multi-dimensional mechanism that may involve modulation of the NF-κB inflammatory axis, rebalancing of gut microbiota structure, and remodeling of microbial metabolic function. These findings provide preliminary evidence for a potential microbe–drug interaction strategy for CG, though causal relationships require further mechanistic validation.

## 1. Introduction

Chronic gastritis (CG) is a common digestive system disease characterized by chronic inflammation of the gastric mucosa. Its core pathological features include inflammatory cell infiltration, intrinsic gland atrophy and reduction, and possible intestinal metaplasia or dysplasia [1,2]. CG has a high global prevalence: the World Health Organization (WHO) statistics indicate that it affects approximately half of the world’s population, constituting a substantial public health burden [3]. There are currently no definitive curative measures for CG [4]. Treatment primarily involves symptomatic management using prokinetic agents, acid suppressants, and gastric mucosal protectants. However, long-term use is frequently associated with adverse reactions and a high rate of recurrence [5], making it difficult to effectively stop disease progression and potentially increasing the risk of gastric cancer [4,6]. According to statistics, millions of individuals die annually from cancers and ulcerative complications related to CG [7,8].

Traditional Chinese medicine (TCM) demonstrates unique advantages in the prevention and treatment of CG. Its diagnostic and therapeutic concepts of “treatment based on syndrome differentiation” and “holistic regulation” exhibit significant efficacy in eliminating causative factors, alleviating clinical symptoms, and improving gastric mucosal pathological morphology, thereby holding promise for delaying disease progression and reducing gastric cancer incidence [9,10]. Although there is no specific disease name corresponding to CG in TCM, based on its core clinical manifestations such as epigastric fullness and distension, pain, acid reflux, belching, nausea, and vomiting [5], it can be categorized under TCM disease patterns such as Wei Wan Tong (epigastric pain), Pi Man (fullness and stuffiness), Cao Za (gastric upset), and E Ni (hiccough) [11]. The pathogenesis of this disease is complex, often arising from factors such as external pathogens invading the stomach, internal damage caused by diet, emotional disorders, and inherent spleen deficiency. These factors lead to spleen and stomach dysfunction, hindering transportation and transformation in the middle energizer and disrupting the ascending and descending functions, subsequently generating pathological consequences such as Qi stagnation, dampness obstruction, and blood stasis, thereby forming a pattern of deficiency in origin and excess in manifestation [12]. The spleen and stomach both reside in the middle energizer. The spleen governs transportation, transformation, and raising the clear, whereas the stomach governs reception and descending the turbid. The two cooperate in reception and transportation, and their ascendance and descendance are interdependent, jointly maintaining the stomach’s physiological state of “discharging without storing, being replete but not full” [13]. TCM treatment frequently begins by harmonizing cold and heat, Qi and blood, and Yin and Yang [9].

Wenweishu Capsules (WWS) are a Chinese patent medicine widely employed in clinical settings to treat CG. Its ingredients include Codonopsis Radix (Dang Shen), Aconiti Lateralis Radix Praeparata (Fu Zi), Astragali Radix (Huang Qi), Cinnamomi Cortex (Rou Gui), Dioscoreae Rhizoma (Shan Yao), Cistanches Herba (Rou Cong Rong), Citri Reticulatae Pericarpium (Chen Pi), Atractylodis Macrocephalae Rhizoma (Bai Zhu), Psoraleae Fructus (Bu Gu Zhi), Crataegi Fructus (Shan Zha), Mume Fructus (Wu Mei), and Amomi Fructus (Sha Ren) [14]. In this formula, Aconiti Lateralis Radix Praeparata, Cinnamomi Cortex, and Cistanches Herba warm and tonify kidney Yang, dispel cold, and relieve pain; Codonopsis Radix, Astragali Radix, Atractylodis Macrocephalae Rhizoma, and Dioscoreae Rhizoma strengthen the spleen and augment Qi, thereby protecting the middle energizer; Amomi Fructus and Citri Reticulatae Pericarpium regulate Qi and harmonize the middle, energize the spleen, and promote appetite; Mume Fructus and Crataegi Fructus, which are sour and sweet, generate Yin, promote digestion, and eliminate food stagnation [15,16]. The formula as a whole achieves the therapeutic effects of warming the middle and nourishing the stomach, while promoting Qi movement and relieving pain. Clinical research has confirmed that when WWS is combined with Western medicine in the treatment of gastritis and gastric ulcers, it can significantly improve therapeutic efficacy [17]. Basic experiments have further indicated that this medicine can effectively reduce gastric juice pH in rat models, enhance pepsin activity, and improve gastric mucosal histopathological damage [18].

In recent years, the role of the gut microbiota as a “hidden organ” in human health and disease has attracted increasing attention [19]. The gut harbors a vast and complex community of microorganisms, which have formed a mutually beneficial symbiotic relationship with the host through long-term co-evolution [20,21]. The stability of the microbial community structure is crucial for maintaining host internal environment homeostasis, and its disruption (dysbiosis) has been demonstrated to be an initiating or promoting factor in the pathogenesis of various diseases [22,23]. Alterations in the composition and quantity of the microbiota can affect intestinal barrier function and local immunity through mechanisms such as microbial metabolites and immune regulation, thereby participating in disease processes [24,25,26]. Decreased microbial diversity has become a hallmark of various disease states, including inflammation and cancer [27]. Studies have indicated that the diversity of the gut microbial community in patients with colorectal cancer is significantly lower than that in healthy individuals [27,28]. Furthermore, gut dysbiosis is closely associated with various metabolic diseases such as obesity, non-alcoholic fatty liver disease, and insulin resistance in type 2 diabetes [29,30,31].

As the initial organ of the digestive tract, the stomach also possesses its own unique microbial community, predominantly composed of Proteobacteria, Firmicutes, Bacteroidetes, Actinobacteria, and Fusobacteria [32,33]. Research has indicated that Helicobacter pylori infection can significantly alter gastric microecology, with one characteristic change being an abnormal increase in the abundance of Lactobacillus [34]. Pathological changes in the gastric mucosa affect its digestive function and local microenvironment, which may subsequently influence the downstream gut microbiota [32,35]. Therefore, investigating the association between CG and gut microbiota dysbiosis, as well as the regulatory effects of effective interventions on the microbiota, is of considerable scientific significance. This study aims to observe changes in the structure and diversity of the gut microbiota in a CG rat model and evaluate whether WWS intervention can correct this dysbiosis, thereby providing new experimental evidence for elucidating this formula’s mechanism of action.

## 2. Results

### 2.1. Description of General Condition

Throughout the experimental period, rats in the blank control (BC) group exhibited a good mental state, rapid responses, white and glossy fur, and normal behavior. Rats in the model control (MC) group began to exhibit messy, sallow, and dull fur, a tendency to huddle together, dry or loose stools, decreased food and water intake, quietness and lethargy, sluggish responses, and reduced activity after 6 weeks of modeling, with symptoms gradually worsening over time. After treatment with WWS, by week 3 of treatment, the mental state and food intake gradually improved, and the huddling behavior decreased. By the end of week 4 of treatment, the fur appeared clean, neat, and glossy.

### 2.2. Analysis of Changes in Food Intake and Body Weight

Throughout the experimental period, changes in food intake and body weight of rats in each group exhibited stage-specific characteristics. In the early stage of modeling (weeks 0–4), the food intake of all groups remained relatively stable (20.29 ± 2.12 g/rat/day), and body weight continued to increase (2.37 ± 0.72 g/rat/day), with no significant differences between groups. Starting from week 5, the food intake of rats in the MC and DT groups began to decrease (15.43 ± 3.08 g/rat/day), and the rate of body weight gain also declined (1.64 ± 0.84 g/rat/day), although this did not represent a statistically significant difference compared with the BC group. By the end of week 8, the food intake of the MC and DT groups decreased further, and body weight gain was significantly lower than that of the BC group (*p* < 0.05). At week 12, which was the end of the modeling period, the food intake of the MC and DT (drug treatment) groups decreased markedly, and body weight gain was significantly lower than that of the BC group (*p* < 0.01) (Table 1 and Table 2). During the drug treatment period (weeks 13–16), the food intake of BC group rats remained stable, whereas the MC group continued to decline and exhibited intermittent feeding behavior. In contrast, the DT group, under the intervention of WWS, demonstrated a gradual recovery in food intake. By week 16, the food intake of the DT group recovered to 25.77 ± 4.04 g/rat/day, and the average body weight gain reached 2.01 ± 0.82 g/rat/day, which significantly differed from the MC group (*p* < 0.05) but not the BC group. These results indicate that WWS intervention effectively improves feeding behavior and promotes weight recovery in CG model rats, suggesting its therapeutic effect on gastritis-related clinical symptoms.

### 2.3. Macroscopic Observation of Changes in Rat Gastric Tissue

Macroscopic observation showed that the gastric tissue of rats in the BC group exhibited deep and regular gastric mucosal folds, a deep red color, and abundant mucus covering the surface, with no edema or bleeding spots observed. Microscopically, the gastric mucosal gland structure was clear and neatly arranged, with only a small number of scattered inflammatory cells infiltrating the lamina propria. Rats in the MC group showed significantly dilated gastric cavities, thinner gastric walls, sparse and superficial folds, and pale gastric mucosa. Microscopic pathological changes were pronounced, manifesting as dilated gastric mucosal glands that were sparse and in disordered arrangement, irregular arrangement of epithelial cells accompanied by necrosis and exfoliation, and destruction of gastric pit structures. In the DT group, after WWS intervention, the gastric cavity was more contracted compared with the MC group, the gastric wall was thicker, folds were deeper, and the mucosal color appeared reddish. The microscopic structure was markedly improved, with the gland structure largely clear, the arrangement tending to be orderly, and epithelial cell organization also more regular. These pathomorphological changes indicate that WWS effectively improves inflammatory responses and structural damage in the gastric tissue of CG rats, as shown in Figure 1.

### 2.4. Analysis of Serum IL-6 and TNF-α Levels

IL-6 and TNF-α are key cytokines involved in mediating inflammatory responses. ELISA results showed that, compared with the BC group, the levels of both IL-6 and TNF-α in the serum of MC group rats were significantly increased (*p* < 0.05). After WWS intervention, the levels of these two inflammatory cytokines in the DT group were significantly reduced, showing a statistically significant difference compared with the MC group (*p* < 0.05), as shown in Table 3. These results indicate that WWS may alleviate local inflammation by downregulating the expression of IL-6 and TNF-α in the serum of CG rats. The specific mechanism underlying this effect requires further investigation.

### 2.5. Immunohistochemical Analysis of RelA and IκBα in Gastric Antrum Tissue

RelA (i.e., NF-κB p65) is a key transcription factor involved in inflammatory responses. IκBα is its inhibitory protein, binding to RelA under resting conditions and preventing its nuclear translocation. When IκBα is degraded, RelA translocates into the nucleus and initiates the transcription of inflammatory genes. However, it is important to note that total IκBα protein levels do not always inversely correlate with NF-κB pathway activity. In certain chronic inflammatory settings, compensatory upregulation of total IκBα may occur due to NF-κB-driven transcriptional feedback, while the phosphorylation status of IκBα (p-IκBα) is the critical determinant of NF-κB activation [36]. IHC staining results (Figure 2) showed that RelA protein, appearing as brownish granules, was primarily localized in the nucleus, and nuclear translocation was clearly observed in the MC group. The IκBα protein appeared as brownish-yellow diffuse granules distributed in the cytoplasm and/or nucleus. The number of positive granules and the staining density for both RelA and IκBα in the MC group were significantly increased compared with the BC group. After WWS intervention, the expression levels of both proteins decreased in the DT group. ImageJ 1.5.4 software IOD analysis further showed that the protein expression levels of RelA and IκBα in the gastric tissue of the MC group were significantly higher than those in the BC group (*p* < 0.05), whereas the expression levels in the DT group were significantly lower than those in the MC group, with a statistically significant difference (*p* < 0.05), as shown in Table 4.

### 2.6. Alpha Diversity Analysis

Alpha diversity analysis based on 16S rRNA sequencing results indicated significant differences in species richness and community structure of the gut microbiota among groups. Specifically, the species richness (Chao1 index) of the MC group was significantly lower than that of the BC group (*p* < 0.05), showing a decrease of 9.7%, which confirms that the model successfully induced microbiota dysbiosis. After WWS intervention, the Chao1 index of the DT group increased by 5.4% compared with the MC group, demonstrating a recovery trend. In terms of community structure, the Simpson index of the MC group was lower than that of the BC group, suggesting reduced dominance and structural instability of the microbiota. In contrast, the Simpson index of the DT group was significantly higher than that of the MC group (*p* < 0.01), indicating that drug intervention promoted the establishment of dominant bacterial populations and stabilized the community structure. The Coverage index for all samples was >0.99, confirming sufficient sequencing depth and reliable data. In summary, WWS may improve the gut microecology of CG model rats by increasing microbiota richness and optimizing community structure, as shown in Figure 3.

### 2.7. Beta Diversity Analysis

Based on the abundance data of the three groups (BC, MC, DT) at the level of 10 differential bacterial genera, principal component analysis (PCA) was performed to assess the overall differences in microbiota structure. PCA employed standardized processing (mean = 0, variance = 1) to eliminate the influence of variable scales. The analysis results (Figure 4A) showed that the first two principal components (PC1 and PC2) together explained 53% of the total variance (PC1: 29.9%, PC2: 23.1%), indicating that these two components effectively captured the major patterns of variation in the data. The PCA score plot demonstrated a partial separation trend among the three groups on the PC1–PC2 plane (Figure 4A). Samples from the BC group were primarily concentrated in the positive PC1 axis region, indicating a relatively stable microbiota structure. MC group samples were relatively concentrated and partially overlapped with the BC group, but overall shifted toward the negative PC1 axis, suggesting that model induction may induce microbiota disturbance. DT group samples were markedly scattered around the intersection area of the positive and negative PC1 axes, between the BC and MC groups, indicating that drug intervention altered the microbiota composition of the model group toward that of the blank control group.

The clustering heatmap of the genus-level community data is shown in Figure 4B. The samples were clearly divided into two main clusters: the first cluster contained all MC group samples, whereas the second cluster contained BC and DT group samples, with BC and DT group samples forming their own subclusters. This pattern indicates significant differences in microbial community structure between the MC group and the BC and DT groups, while the DT group exhibited high similarity to the BC group. Specifically, the MC group showed lower abundance of the beneficial bacterium the *Lachnospiraceae NK4A136 group* but higher abundance of potential pathobionts such as *Lactobacillus* and *Akkermansia*. Conversely, the BC and DT groups exhibited lower abundances of *Lactobacillus* and *Akkermansia*. Clustering of bacterial taxa further showed that beneficial bacteria associated with short-chain fatty acid production (*Lachnospiraceae NK4A136 group*) clustered in the BC and DT groups, whereas bacteria associated with pathogenesis (*Lactobacillus* and *Akkermansia*) clustered in the MC group. Notably, DT group samples clustered closely with BC group samples, indicating that drug treatment effectively reversed the model-induced changes in the microbiota and restored a microbial composition similar to that of the control group. This result highlights the protective effect of drug treatment and provides microbiological evidence for related therapeutic strategies.

### 2.8. Species Composition Analysis

#### 2.8.1. Phylum-Level Microbiota Composition Analysis

The present study investigated the effect of drug intervention on the gut microbiota of model rats at the phylum level through 16S rRNA gene sequencing. Community structure analysis (Figure 5A) showed that the microbial composition of the MC group was markedly altered, characterized by a decrease in the relative abundance of Firmicutes to 69.99% and an increase in Bacteroidetes abundance to 24.21%. The F/B ratio, a widely used indicator of gut microbial dysbiosis, was significantly decreased in the MC group (2.89) compared with the BC group (3.57) (*p* < 0.01). This dysbiotic profile was further accompanied by enrichment of Verrucomicrobia (3.97%) and Actinobacteria (0.57%), as shown in Figure 5B. Notably, treatment with WWS effectively reversed the model-induced microbiota dysbiosis: it increased the F/B ratio to 3.88, suppressed the abnormal proliferation of Verrucomicrobia (0.80%) and Actinobacteria (0.19%), and remodeled the overall community structure to a state not significantly different from the BC group. In summary, WWS restores the dysregulated gut microbial ecosystem toward a normal state by regulating the balance of core phyla, which may represent one of the important mechanisms underlying its therapeutic effect.

#### 2.8.2. Genus-Level Composition Analysis

Statistical analysis of the intestinal genus-level microbiota structure of the BC, MC, and DT groups (Figure 6A) showed that, compared with the BC group, the MC group exhibited significant differences at the genus level (*p* < 0.05). Among them, the *Lachnospiraceae NK4A136 group* (Figure 6B), an important, beneficial bacterium, showed an abundance in the MC group (10.25% ± 6.62%) that was significantly lower than that in the BC group (16.07% ± 8.24%). In contrast, the abundances of the potential pathobionts *Lactobacillus* (MC: 11.39% ± 5.09% vs. BC: 7.03% ± 4.51%, Figure 6C), *Bacteroides* (MC: 6.46% ± 4.52% vs. BC: 4.22% ± 1.75%, Figure 6D), and *Akkermansia* (MC: 3.97% ± 2.67% vs. BC: 1.29% ± 2.29%, Figure 6E) were all increased in the MC group. After WWS intervention, the DT group reversed this dysbiotic state: the abundance of the *Lachnospiraceae NK4A136 group* increased to 28.34% ± 6.32% (significantly higher than the MC group, *p* < 0.01), whereas the abundances of *Lactobacillus* (4.55% ± 1.74%), *Bacteroides* (2.68% ± 1.07%), and *Akkermansia* (0.30% ± 0.15%) were all significantly lower than those in the MC group (*p* < 0.01). These results suggest that the sodium deoxycholate–ammonia water–ethanol–irregular feeding method triggers local gastric mucosal inflammation and promotes CG through the gut–stomach axis by inducing gut microbiota imbalance, whereas WWS may exert therapeutic effects on CG by promoting the proliferation of beneficial bacteria and inhibiting the overgrowth of potential pathobionts.

### 2.9. Functional Prediction Analysis

PICRUSt functional prediction based on 16S rRNA sequences is shown in Figure 7. The 15 samples were involved in a total of 45 KEGG level-2 metabolic pathways. The functions of the rat gut microbiota were primarily concentrated in metabolic pathways such as amino acid metabolism, carbohydrate metabolism, metabolism of cofactors and vitamins, biosynthesis of secondary metabolites, biosynthesis and metabolism of glycerolipids, energy metabolism, lipid metabolism, metabolism of terpenoids and polyketides, and biodegradation and metabolism of xenobiotics. It is inferred that the diverse metabolic enzyme systems synthesized by rat gut bacteria enhance their metabolic rate and increase the diversity and complexity of microbial metabolism.

Through analysis of 16S rRNA gene functional prediction data, the present study revealed gut microbiota functional dysregulation induced by the CG model and the intervention effect of WWS at the microbial functional level. Compared with the BC group, the gut microbiota of model group rats exhibited widespread functional disturbances: core substance and energy metabolism pathways, including amino acid metabolism (Figure 8A, −3.1%), carbohydrate metabolism (Figure 8B, −3.5%), and energy metabolism (Figure 8C, −3.1%), were significantly downregulated. Meanwhile, pathways related to xenobiotics biodegradation and metabolism (Figure 8D, +6.1%) and bacterial infectious diseases (Figure 8E, +5.2%) were abnormally enhanced, suggesting that the microbiota in the model group may exist in a pro-inflammatory and metabolic stress state. Additionally, the sharp decline in cell motility (Figure 8F, −20.0%) also indicated impaired colonization and interaction capacities of the microbiota.

Drug intervention effectively reversed the functional disturbances outlined above. Drug treatment significantly restored core metabolic capacities, bringing the abundances of carbohydrate metabolism (+3.9%) and amino acid metabolism (+3.8%) back toward normal levels. Simultaneously, it suppressed the overexpression of harmful functions, such as pathways related to bacterial infectious diseases (−3.4%) and xenobiotics metabolism (−9.8%), demonstrating potential anti-inflammatory effects and a reduction in metabolic burden. WWS also enhanced the fundamental physiological functions of the microbiota, showing notable promoting effects on membrane transport (+8.9%), signal transduction (+4.2%), and cell motility (+44.8%). Ultimately, the functional profile of the microbiota in the DT group was highly similar to that of the BC group (differences in most key pathways were within ±1%).

Thus, it can be concluded that WWS does not act on a single target but rather modulates gut microbiota functions in a multidimensional and systematic manner, including restoring core metabolism, inhibiting harmful functions, and enhancing basic physiological activities. Through these coordinated effects, the dysregulated gut microbial ecosystem is remodeled toward a healthy and balanced state, which may represent one of the important mechanisms underlying its therapeutic effect in CG.

### 2.10. Correlation Analysis Between Differential Bacterial Phyla/Genera and Inflammatory Factors

To explore the association between gut microbiota and systemic inflammation, Pearson correlation analysis was performed between four differential bacterial phyla and four inflammation-related factors across the three groups. The results revealed several statistically significant associations, suggesting the potential pro- or anti-inflammatory functions of specific phyla. The results are shown in Figure 9. Firmicutes exhibited clear anti-inflammatory properties, with their abundance showing a highly significant positive correlation with the NF-κB endogenous inhibitory protein IκBα (r = 0.73, *p* < 0.01), and a significant negative correlation with the pro-inflammatory factor TNF-α (r = −0.64, *p* < 0.05). This finding indicates that the proliferation of Firmicutes was accompanied by a significant increase in IκBα levels, thereby effectively inhibiting the expression of the pro-inflammatory factor TNF-α (the proportions of Firmicutes in the BC and DT groups were 75.8% and 77.9%, respectively, whereas in the MC group it was 69.9%). In contrast, Bacteroidetes showed strong pro-inflammatory potential, with its abundance significantly positively correlated with TNF-α and IL-6 levels (TNF-α: r = 0.69, *p* < 0.01; IL-6: r = 0.71, *p* < 0.001), corresponding to the highest level of IκBα in MC (95.3%) (the proportions in the BC and DT groups were 70.2% and 47.9%, respectively). In summary, this study preliminarily identified the potential key roles of Firmicutes and Bacteroidetes (r = −0.66, *p* < 0.01) in inflammation regulation, providing an important theoretical basis for subsequent in-depth investigation of their causal mechanisms and targeted regulation.

Correlation analysis between differential gut bacterial genera and inflammation-related indicators is shown in Figure 10. The *Lachnospiraceae NK4A136 group* exhibited the most prominent anti-inflammatory properties. Its abundance was not only significantly positively correlated with the level of the NF-κB pathway inhibitory protein IκBα (r = 0.78, *p* < 0.01), but also negatively correlated with the concentrations of key pro-inflammatory factors TNF-α and IL-6 (TNF-α: r = −0.75, *p* < 0.01; IL-6: r = −0.62, *p* < 0.05), suggesting that it may inhibit the expression of TNF-α and IL-6 via the NF-κB pathway by elevating IκBα, thereby alleviating inflammation. In contrast, *Bacteroides* showed a pro-inflammatory tendency, with the abundance significantly positively correlated with TNF-α and IL-6 levels (TNF-α: r = 0.97, *p* < 0.01; IL-6: r = 0.65, *p* < 0.05). These findings provide potential microbial targets for the targeted regulation of gut microbiota in the management of inflammatory diseases and highlight the crucial role of the balance between the *Lachnospiraceae NK4A136 group* and *Bacteroides* in maintaining intestinal immune homeostasis.

## 3. Discussion

CG is a complex multifactorial disease, and its pathogenesis has expanded from traditional local gastric mucosal inflammation to encompass systemic regulatory imbalance of the gut–stomach axis [37,38,39]. Based on this pathological concept, the present study successfully established a CG rat model using composite factors and systematically explored the multi-target synergistic mechanism of WWS in treating CG at multiple levels, including the whole organism, tissue, cell, and microbiome.

### 3.1. Composite Factor Modeling Effectively Simulates the Complex Pathological Characteristics of Clinical CG

The sodium deoxycholate–ammonia water–ethanol–irregular feeding composite modeling method used in this study integrated various pathogenic factors such as chemical injury (simulating bile reflux and a high-ammonia environment), physical stimulation (ethanol), and biological rhythm disturbance (irregular feeding). This multifactorial intervention mode closely resembles the clinical characteristics of CG, which often involves the interaction of factors such as poor dietary habits, stress, and Helicobacter pylori infection [40]. Experimental results showed that this model not only successfully induced typical pathological changes in the gastric mucosa, including inflammatory cell infiltration and glandular structural disorder, but also caused systemic manifestations such as delayed weight gain, decreased food intake, and withered fur. These findings are consistent with the results reported by Ji et al. [41], who used similar methods, thereby validating the reliability of the model construction and providing a solid foundation for subsequent evaluation of drug intervention.

### 3.2. WWS Alleviates Gastric Inflammation by Inhibiting the NF-κB Pathway

This study validated that WWS significantly improves gastric lesions in CG model rats, and its effects are associated with the NF-κB signaling pathway. Experimental results showed that WWS not only significantly reduced the levels of key pro-inflammatory factors such as TNF-α and IL-6 in the serum of CG model rats, but also downregulated the overexpression of the NF-κB key subunit RelA (p65) and its inhibitory protein IκBα in gastric tissue. The NF-κB pathway, as the “core switch” of the body’s inflammatory response, plays a central role in the occurrence and development of CG [42]. Upon external stimulation, IκBα is phosphorylated and degraded, releasing its inhibition of RelA and allowing it to translocate into the nucleus to initiate transcription of inflammatory genes such as TNF-α and IL-6, thereby forming an inflammatory cascade amplification effect [43]. However, we acknowledge that total IκBα levels alone do not fully reflect NF-κB pathway activity, as compensatory upregulation of IκBα may occur in chronic inflammatory states due to NF-κB-driven transcriptional feedback [36]. The phosphorylation status of IκBα (p-IκBα) is the critical determinant of NF-κB activation, and future studies should assess the p-IκBα/total IκBα ratio to more accurately evaluate pathway dynamics. The results of the present study indicate that WWS intervention is associated with altered IκBα expression and modulation of RelA nuclear translocation, which may contribute to the regulation of the NF-κB pathway. This finding provides molecular biological evidence supporting the clinical anti-inflammatory efficacy of WWS, though the causal relationship requires further mechanistic validation.

### 3.3. WWS Balances the Gut–Stomach Axis Function by Remodeling the Gut Microbiota

A key finding of this study is the observation that WWS treatment coincides with the restructuring of the gut microbiota ecology in CG rats. Recent studies have confirmed that gut microbiota dysbiosis actively participates in and exacerbates gastric inflammation through immune, metabolic, and neural pathways [44,45]. 16S rRNA sequencing analysis showed that CG model rats exhibited significant gut microecological imbalance, and WWS intervention effectively reversed this trend.

WWS promoted the proliferation of beneficial bacteria. After WWS treatment, the abundance of butyrate-producing bacteria (*Lachnospiraceae NK4A136 group* and *Roseburia*) was significantly increased. Butyrate produced by these bacteria during the fermentation of dietary fiber has been demonstrated to possess strong anti-inflammatory properties and to maintain intestinal barrier integrity [46]. It can indirectly inhibit the NF-κB pathway through mechanisms such as the inhibition of histone deacetylases and activation of G-protein coupled receptors [47], which is consistent with the observed reductions in inflammatory factor levels and inhibition of the NF-κB pathway in the DT group.

WWS effectively inhibited the overgrowth of potential pathobionts such as *Lactobacillus*, *Bacteroides*, and the mucus-degrading bacterium *Akkermansia*. Under disease conditions, the abnormal increase in *Akkermansia* may be associated with erosion of the intestinal mucus layer, thereby increasing the risk of pathogen translocation [48]. However, we acknowledge that no direct experimental evidence (e.g., Western blotting or immunofluorescence for tight junction proteins such as ZO-1, Occludin, and Claudin-1, or histological assessment of intestinal tissue) is currently available to support the hypothesis that WWS improves intestinal barrier function. This represents an important limitation of the present study.

### 3.4. Microbiota Function Prediction and Correlation Analysis Reveal the Action Network

PICRUSt functional prediction showed that WWS intervention upregulated core microbial activities such as carbohydrate metabolism and energy metabolism, while downregulating pathways related to bacterial infections. This finding indicates that WWS altered the microbiota structure and optimized its functional state, shifting it from a pro-inflammatory and metabolically disordered condition toward an anti-inflammatory and metabolically balanced state. Spearman correlation analysis further provided direct supporting evidence: the pro-inflammatory factors TNF-α and IL-6, as well as the signaling protein RelA, were significantly positively correlated with potential pathobionts and significantly negatively correlated with the beneficial bacterium *Lachnospiraceae* NK4A136 group. These results strongly suggest that the specific bacteria regulated by WWS may function as key mediators of its systemic anti-inflammatory effects, constituting the core link of the microbiota–immune–gastric mucosa regulatory axis.

### 3.5. Study Limitations and Prospects

The present study has several important limitations that must be acknowledged:

(1) **Observational nature**: Although correlation analysis suggests a close association between microbiota alterations and inflammation alleviation, this study cannot establish a causal relationship between gut microbiota changes and gastric mucosal protection. Fecal microbiota transplantation (FMT) from WWS-treated animals into recipient animals with chronic gastritis, microbiota depletion using antibiotics followed by WWS treatment, or experiments using germ-free animals would be required to establish a causal role of the gut–gastric axis in WWS-mediated therapeutic effects [49].
(2) **Lack of intestinal barrier assessment**: An important limitation is the lack of direct evaluation of intestinal barrier integrity and intestinal inflammation. No experimental evidence—such as Western blotting or immunofluorescence for tight junction proteins (ZO-1, Occludin, Claudin-1) or histological assessment of intestinal tissue—is currently available to support the hypothesis that WWS strengthens intestinal barrier function.
(3) **Metabolite validation**: Changes in the in vivo concentrations of specific microbiota metabolites (such as butyrate) and their direct functional evidence still require further confirmation through metabolomics studies.
(4) **IκBα phosphorylation status:** The present study measured total IκBα protein levels by immunohistochemistry but did not assess the phosphorylation status of IκBα (p-IκBα). Future studies should evaluate the p-IκBα/total IκBα ratio to more accurately assess NF-κB pathway activation dynamics [36].

Despite these limitations, this study provides preliminary evidence for the association between WWS treatment, gut microbiota restructuring, and gastric inflammation improvement, offering a foundation for future mechanistic investigations.

## 4. Materials and Methods

### 4.1. Experimental Animals and Treatment

After an acclimatization period, 15 Wistar rats aged 6–8 weeks (from the SPF-level Experimental Animal Center of Gansu University of Chinese Medicine, Lanzhou, China) were randomly assigned to three groups (n = 5): the blank control (BC), model control (MC), and drug treatment (DT) groups. The CG model was established with reference to and modifications based on the literature [50]. Rats in the MC and DT groups were administered 20 mmol/L sodium deoxycholate (Shanghai Macklin Biochemical Technology Co., Ltd., Shanghai, China) by gavage daily (2 mL/rat); allowed free access to 0.03% ammonia water (Shanghai Macklin Biochemical Technology Co., Ltd., Shanghai, China); and gavaged with 60% ethanol (Shanghai Macklin Biochemical Technology Co., Ltd., Shanghai, China) every 3 days (2 mL/rat). Simultaneously, a dietary rhythm was implemented that consisted of 2 days of satiety and 1 day of fasting (water allowed). This intervention continued for 12 consecutive weeks. Starting from week 13, drug treatment commenced. The BC and MC groups received an equal volume of physiological saline by gavage daily, whereas the DT group received 0.38 g/kg/d WWS suspension (Hefei Huaren Shenlu Pharmaceutical Co., Ltd., Hefei, China) by gavage for 4 weeks. The dosage of 0.38 g/kg/d was calculated based on the equivalent dose conversion between humans and rats using the body surface area normalization method: human clinical dose (1.5 g/60 kg/day) × conversion factor (6.2)/60 kg ≈ 0.38 g/kg/day [51]. Throughout the experimental period, all rats had free access to standard feed (containing 20.0% protein and 64.9% carbohydrate). Food intake was recorded daily at 9:00 AM by weighing the remaining chow. Daily food consumption per rat was calculated as (food provided − food remaining)/number of rats per cage. The average daily food intake over the 4-week treatment period was used for statistical analysis. All rats were deeply anesthetized at the experimental endpoint via intraperitoneal injection of a 1% sodium pentobarbital solution (Shanghai Macklin Biochemical Technology Co., Ltd., Shanghai, China) at a dose of 45 mg/kg body weight. Once both the corneal reflex and toe-pinch reflex were completely absent, blood was collected via either the abdominal aorta or cardiac puncture. Immediately after blood collection, euthanasia was performed by intraperitoneal injection of a triple dose of sodium pentobarbital (135 mg/kg). Cessation of respiration was followed by cervical dislocation as a secondary confirmation of death. All procedures were approved by the Animal Ethics Committee of Yili Normal University (Approval No. 2024-06) and conducted in accordance with the ARRIVE guidelines to minimize animal suffering. This study is reported in accordance with ARRIVE guidelines (https://arriveguidelines.org).

### 4.2. General Condition Observation and Body Weight Monitoring

During the experiment, the mental state, spontaneous activity, fur color, lips, tongue appearance, ear and tail color, and stool characteristics of rats in each group were observed and recorded daily. At fixed time points each week, after fasting for 24 h, rat body weight was measured, and food intake was recorded.

### 4.3. Gastric Tissue Pathological Examination

After the final administration, rats were anesthetized and dissected, and the entire stomach was removed. Gastric morphology, color, texture, and mucosal folds were observed macroscopically. Tissue samples from the gastric antrum were fixed in 4% paraformaldehyde (Shanghai Macklin Biochemical Technology Co., Ltd., Shanghai, China) for 24 h. After gradient dehydration and paraffin embedding, 4 μm-thick sections were prepared. Hematoxylin and eosin (HE) staining (Beijing Solarbio Science & Technology Co., Ltd., Beijing, China) was performed, and pathological changes including gastric mucosal inflammation, atrophy, and metaplasia were observed under a light microscope. Pathological assessment was conducted according to the criteria of the “Expert Consensus on TCM Diagnosis and Treatment of Chronic Gastritis” [52].

### 4.4. Serum Inflammatory Cytokine Assay

Blood was collected via cardiac puncture, allowed to stand at room temperature for 2 h, and then centrifuged at 2000 rpm for 10 min. The supernatant serum was aliquoted into EP tubes. The levels of TNF-α and IL-6 in serum were determined using enzyme-linked immunosorbent assay (ELISA) kits (Hangzhou Lianke Biotechnology Co., Ltd., Hangzhou, China), following the manufacturer’s instructions.

### 4.5. Immunohistochemical (IHC) Detection

Paraffin sections of the gastric antrum were subjected to immunohistochemical staining to detect the localization and expression of NF-κB p65 (RelA) and IκBα proteins (Saixin Tong (Shanghai) Biological Reagent Co., Ltd., Shanghai, China). The appearance of brownish-yellow granules in the nucleus or cytoplasm was considered a positive signal. Four random 400× fields were selected for each section, and the integrated optical density (IOD) of the positive area was calculated using ImageJ 1.5.4 software for semi-quantitative analysis.

### 4.6. Gut Microbiota 16S rRNA Sequencing Analysis

Colon contents were collected from the rats, and total fecal DNA was extracted using a commercial kit (Tiangen Biotech Co., Ltd., Beijing, China) according to the manufacturer’s instructions. Using 338F (5′-ACTCCTACGGGAGGCAGCA-3′) and 806R (5′-GGACTACHVGGGTWTCTAAT-3′) as primers, the V3–V4 region of the bacterial 16S rRNA gene was amplified by PCR. After purification of the amplification products, sequencing was performed on the Illumina PE250 platform by Shanghai Ling’en Biotechnology Co., Ltd. Based on barcode sequences and PCR amplification primer sequences, sequencing data for each sample were demultiplexed. After removing barcodes and primer sequences, FLASH software (V1.2.11) was used to merge paired-end reads for each sample, generating Raw Tags. Subsequently, Fastp software (V0.19.4) was used for quality control of the Raw Tags to generate high-quality Clean Tags. Finally, Vsearch software (V2.23) was used to align the Clean Tags with the reference database to detect and remove chimeras, thereby obtaining the final effective data, namely Effective Tags. For the obtained Effective Tags, the DADA2 module in QIIME2 software (V2022.11) was used for denoising, and sequences with an abundance of less than five were filtered out, resulting in the final amplicon sequence variants (ASVs). The classify-sklearn module in QIIME2 was used to compare ASVs with the database to obtain species annotation information corresponding to each ASV. QIIME2 2026.4 software was used to calculate Chao1, Ace, Shannon, and Simpson indices. Unifrac distances were calculated using QIIME2 software, and the ade4 (V1.7-23) and ggplot2 (V3.5.1) packages were used to generate PCoA plots. At the genus level, MetaStat analysis was conducted for comparisons between two groups, and R 4.6.1 software was used for differential testing to obtain *p*-values. Genera with *p* < 0.05 were identified as significantly different taxa between groups.

### 4.7. Microbiota Function Prediction

Based on the obtained ASV data, PICRUSt2 v2.5.3 software [17] was used to predict metagenomic functions. The predicted results were compared with the Clusters of Orthologous Groups (COG) and the Kyoto Encyclopedia of Genes and Genomes (KEGG) databases to obtain abundance information for each functional category (COG, KO, KEGG Pathway), thereby inferring potential functional alterations in the microbiota.

### 4.8. Statistical Analysis

All data are expressed as the mean ± standard deviation. GraphPad Prism 9 and Origin 2021 software were used for statistical analyses. One-way analysis of variance (ANOVA) was applied for comparisons among multiple groups. If the ANOVA results indicated significant differences (* *p* < 0.05), Tukey’s test was subsequently used for pairwise comparisons. *p* < 0.05 was considered statistically significant.

### 4.9. Preprint Statement

A preliminary version of this manuscript has been deposited as a preprint on Research Square under the title “Remodeling of Gut Microbiota Metabolic Function Mediates the Therapeutic Effect of Wenweishu Capsules on the Gut–Gastric Axis in Chronic Gastritis” [53].

## 5. Conclusions

From the holistic perspective of the gut–stomach axis, this study systematically elucidated the multidimensional associations of WWS treatment with inhibition of the NF-κB inflammatory axis, rebalancing of the gut microbiota structure, and remodeling of microbial metabolic functions, which were concurrently observed with improvements in CG gastric lesions and microecological disorders. These findings are observational in nature, and whether the gut microbiota alterations are causally linked to gastric protection requires further mechanistic studies, such as fecal microbiota transplantation or antibiotic depletion experiments. This study expands the understanding of TCM as acting only locally in the stomach by identifying the gut microbiota as a potential key therapeutic target. It provides preliminary scientific evidence for explaining the TCM concept of holistic regulation and suggests a theoretical framework for developing new CG treatment strategies based on bacteria–medicine synergy. However, the causal role of the gut–gastric axis in WWS-mediated therapeutic effects remains to be established.

## Figures and Tables

**Figure 1:**
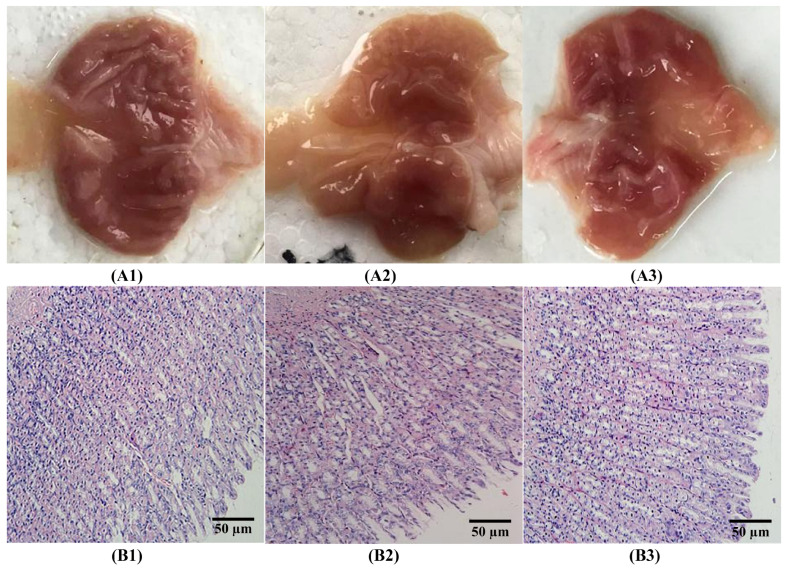
Pathological anatomy and histological staining of rat gastric tissue. (**A**) Gross pathological anatomy images showing the gastric mucosal folds, color, texture, and mucus coverage in the blank control (BC; (**A1**)), model control (MC; (**A2**)), and drug treatment (DT; (**A3**)) groups. (**B**) Hematoxylin and eosin (HE) staining images (magnification, ×200) showing the glandular structure, inflammatory cell infiltration, and epithelial integrity in the BC (**B1**), MC (**B2**), and DT (**B3**) groups. Note: Figure 1(B3) has been replaced with a higher-resolution image. Wenweishu Capsules effectively improved gastric mucosal damage and pathological changes in chronic gastritis rats.

**Figure 2:**
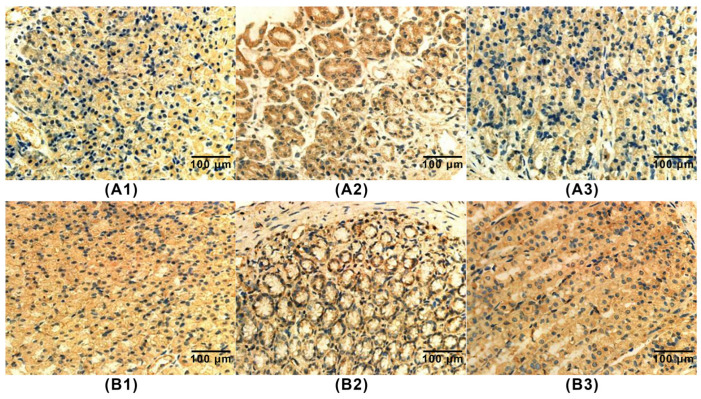
Immunohistochemical detection of RelA (**A**) and IκBα (**B**) protein expression levels in rat gastric antrum tissue (magnification, ×400). RelA protein was localized in the nucleus as brownish granules, and IκBα protein was distributed in the cytoplasm and/or nucleus as brownish-yellow diffuse granules. Images represent the BC, MC, and DT groups. (**A1**) Blank control group; (**A2**) Model control group; (**A3**) Drug treatment group; (**B1**) Blank control group; (**B2**) Model control group; (**B3**) Drug treatment group.

**Figure 3:**
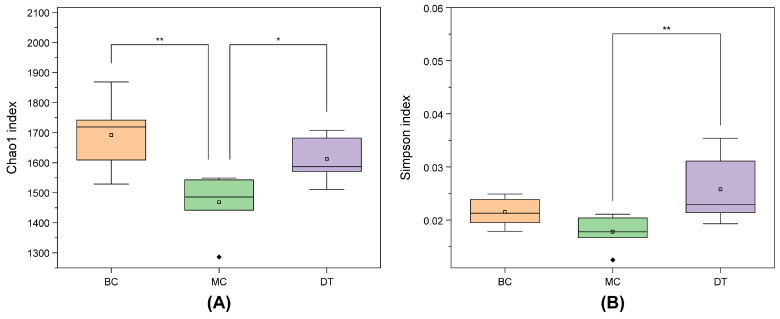
Effects of Wenweishu Capsules on the alpha diversity of gut microbiota in rats with chronic gastritis. (**A**) Chao1 index reflecting species richness. (**B**) Simpson index reflecting community dominance and evenness. Note: The data in (**A**,**B**) have been corrected from the preprint version following re-checking of raw data. * indicates *p* < 0.05 and ** indicates *p* < 0.01, representing significant differences between the three groups (BC, blank control; MC, model control; DT, drug treatment).

**Figure 4:**
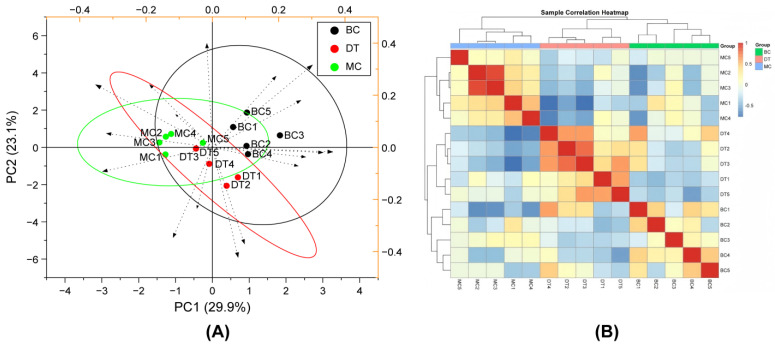
Structural shifts in gut microbiota and the restorative effect of Wenweishu Capsules in chronic gastritis rats. (**A**) Principal component analysis (PCA) score plot based on the abundance of 10 differential bacterial genera, showing the clustering patterns of the BC, MC, and DT groups. PC1 and PC2 represent the first and second principal components, respectively. We confirm that an explanation of the circle colors should be added to the figure caption. Red circle represent the drug treatment group, green circle represent the model control group, and black circle represent the blank control group. (**B**) Hierarchical clustering heatmap based on the relative abundance of 10 differential genera, illustrating the similarity of microbial community structure among samples. The color scale indicates the relative abundance after z-score normalization.

**Figure 5:**
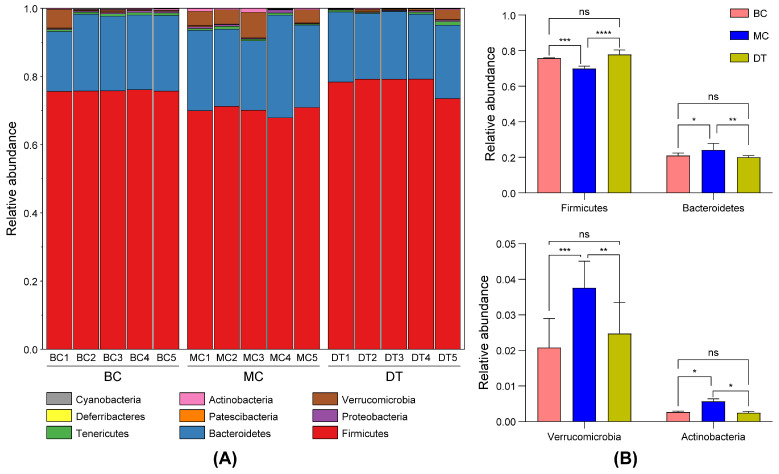
Alterations in the phylum-level gut microbiota structure among the rat groups. (**A**) Relative abundance of the major bacterial phyla (Firmicutes, Bacteroidetes, Verrucomicrobia, Actinobacteria, and others) in the BC, MC, and DT groups. (**B**) Statistical comparison of the differentially abundant phyla (Firmicutes, Bacteroidetes, Verrucomicrobia, and Actinobacteria) and the Firmicutes/Bacteroidetes (F/B) ratio among the three groups. * indicates *p* < 0.05, ** indicates *p* < 0.01, *** indicates *p* < 0.001, and **** indicates *p* < 0.0001. The Firmicutes/Bacteroidetes (F/B) ratios, calculated from the relative abundances of the two phyla shown above, were 3.57 (BC), 2.89 (MC), and 3.88 (WWS), respectively.

**Figure 6:**
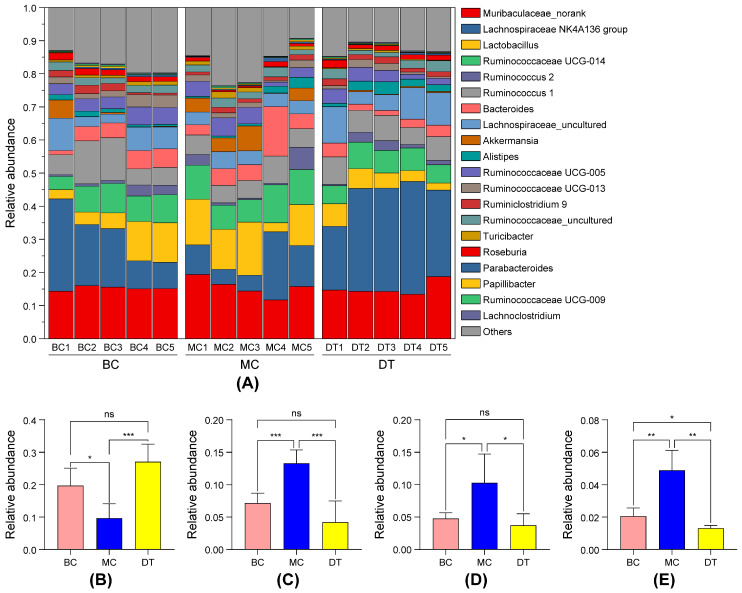
Wenweishu Capsules reverse chronic gastritis-induced dysbiosis at the genus level by modulating specific beneficial and opportunistic pathogenic bacteria. (**A**) The overall structural composition of the gut microbiota at the genus level in the BC, MC, and DT groups. (**B**–**E**) Statistical analysis of the relative abundances of four representative differential genera: (**B**) the beneficial bacterium *Lachnospiraceae NK4A136 group*, (**C**) *Lactobacillus*, (**D**) *Bacteroides*, and (**E**) *Akkermansia*. * indicates *p* < 0.05, ** indicates *p* < 0.01, *** indicates *p* < 0.001.

**Figure 7:**
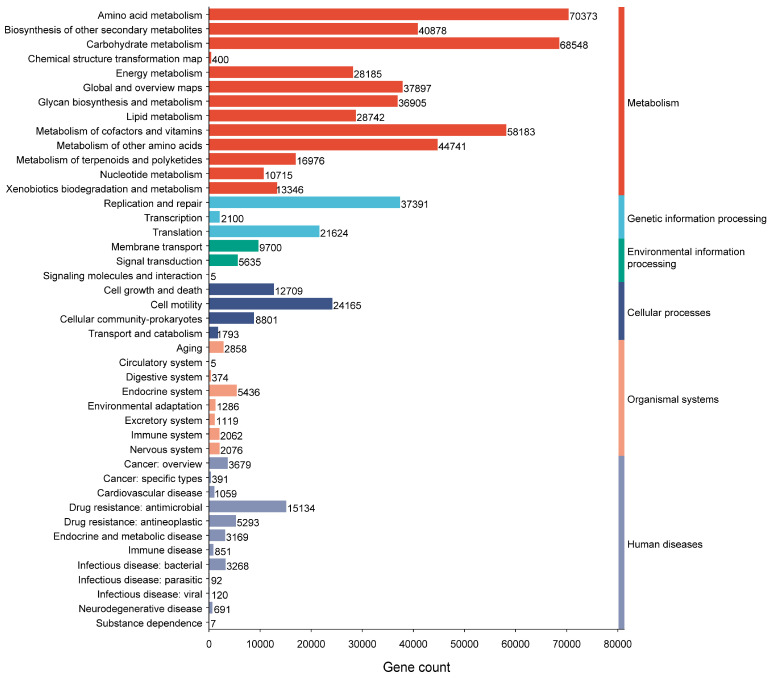
Predictive functional profiling of gut microbiota based on 16S rRNA sequencing using the PICRUSt2 algorithm and the Kyoto Encyclopedia of Genes and Genomes (KEGG) database. The functional profiles show the relative abundance of level-2 KEGG metabolic pathways across the BC, MC, and DT groups, including amino acid metabolism, carbohydrate metabolism, energy metabolism, and others.

**Figure 8:**
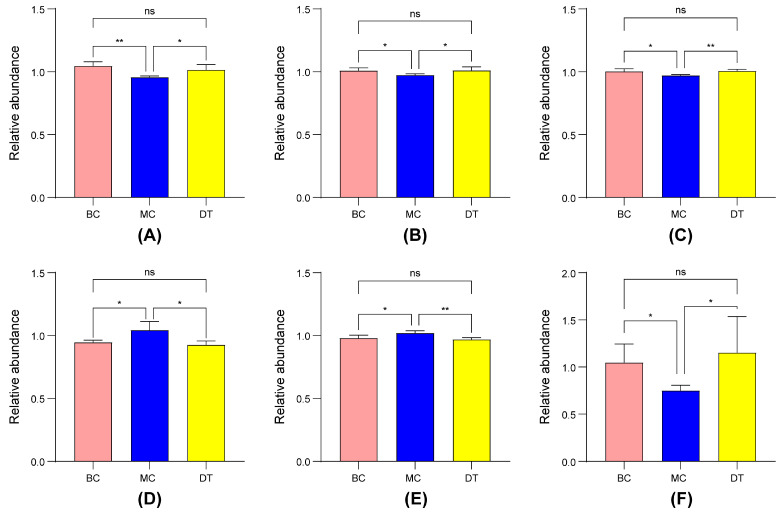
Comparative analysis of KEGG pathway differences between the BC, MC, and DT groups. (**A**) Amino acid metabolism. (**B**) Carbohydrate metabolism. (**C**) Energy metabolism. (**D**) Xenobiotics biodegradation and metabolism. (**E**) Infectious bacterial disease. (**F**) Cell motility. Data are presented as the mean relative abundance ± standard deviation. * indicates *p* < 0.05, ** indicates *p* < 0.01.

**Figure 9:**
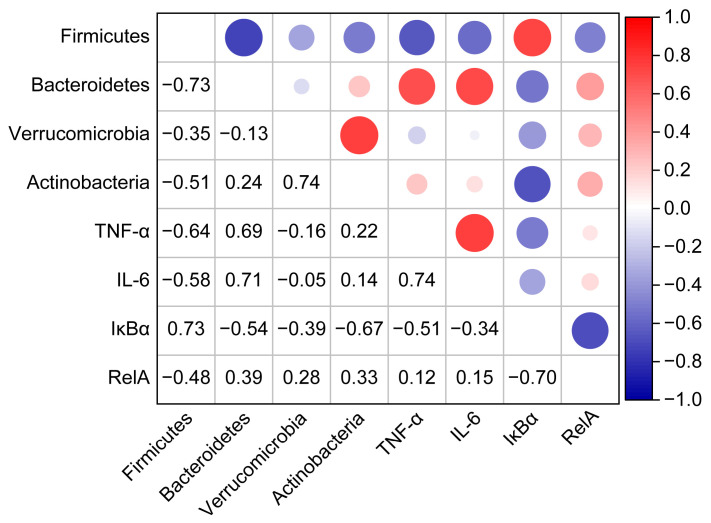
Pearson correlation analysis between differential gut microbiota at the phylum level (Firmicutes, Bacteroidetes, Verrucomicrobia, and Actinobacteria) and host inflammatory factors (TNF-α, IL-6, IκBα, and RelA). Correlation coefficients (r) are shown, where positive values indicate positive correlations and negative values indicate negative correlations.

**Figure 10:**
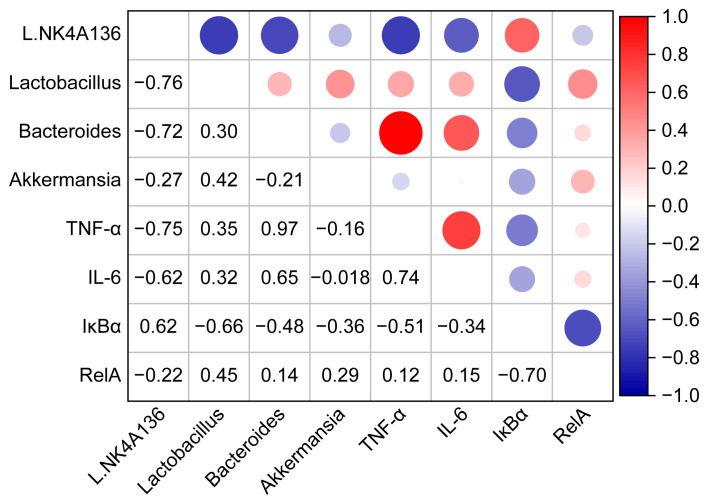
Pearson correlation analysis between differential gut microbiota at the genus level (*Lachnospiraceae NK4A136 group*, *Lactobacillus*, Bacteroides, and *Akkermansia*) and host inflammatory factors (TNF-α, IL-6, IκBα, and RelA). Correlation coefficients (r) are shown, where positive values indicate positive correlations and negative values indicate negative correlations.

**Table 1:** Analysis of the effect of *Wenweishu* capsules on rat body weight.

Group	Body Weight (g)				
	Week 0	Week 4	Week 8	Week 12	Week 16
BC	181.42 ± 34.38	228.72 ± 32.22	264.36 ± 31.47	292.33 ± 55.69	322.20 ± 57.68
MC	181.36 ± 34.15	224.26 ± 37.23	249.85 ± 42.61 ^a^	263.83 ± 53.01 ^a^	292.89 ± 61.49 ^a^
DT	181.25 ± 32.47	224.71 ± 31.39	247.88 ± 41.89 ^a^	265.17 ± 57.41 ^a^	313.26 ± 69.15 ^b^

Each sample was measured in duplicate (technical replicates), and data are presented as mean ± SD of 5 biological replicates. ^a^ represents a comparison with the blank group: *p* < 0.05; ^b^ represents comparison with the model group, *p* < 0.05.

**Table 2:** Analysis of changes in rat daily feed intake.

Group	Feeding Intake (g/Rat/d)			
	Weeks 0–4	Weeks 5–7	Weeks 8–12	Weeks 13–16
BC	18.02 ± 2.33	26.91 ± 4.27	28.91 ± 4.32	29.66 ± 5.09
MC	17.21 ± 2.73	25.17 ± 4.52	23.06 ± 3.78 ^a^	20.37 ± 3.75 ^a^
DT	18.89 ± 1.97	24.89 ± 4.21	24.72 ± 3.59 ^a^	25.77 ± 4.04 ^b^

Each sample was measured in duplicate (technical replicates), and data are presented as mean ± SD of 5 biological replicates. ^a^ represents a comparison with the blank group: *p* < 0.05; ^b^ represents comparison with the model group, *p* < 0.05.

**Table 3:** Effect of *Wenweishu* Capsule on the levels of TNF-α and IL-6 in serum of rats in each group.

Group	Dosage (g/kg)	TNF-α (pg/mL)	IL-6 (pg/mL)
Blank control	Physiological saline	8.91 ± 2.02	26.95 ± 8.52
Model control	Physiological saline	12.26 ± 3.58 ^a^	49.45 ± 16.83 ^a^
Drug treatment	0.38	9.33 ± 1.15 ^b^	34.22 ± 22.93 ^b^

Each sample was measured in duplicate (technical replicates), and data are presented as mean ± SD of 5 biological replicates. ^a^ represents a comparison with the blank group: *p* < 0.05, ^b^ represents comparison with the model group, *p* < 0.05.

**Table 4:** IHC detected RelA and IκBα protein expression levels in gastric antrum tissues of rats in each group.

Group	Dosage (g/kg)	RelA	IκBα
Blank control	Physiological saline	2.17 ± 0.35	2.12 ± 0.33
Model control	Physiological saline	3.28 ± 0.49 ^a^	3.44 ± 0.98 ^a^
Drug treatment	0.38	2.38 ± 0.39 ^b^	2.39 ± 0.82 ^b^

Each sample was measured in duplicate (technical replicates), and data are presented as mean ± SD of 5 biological replicates. ^a^ represents a comparison with the blank group: *p* < 0.05; ^b^ represents comparison with the model group, *p* < 0.05.

## Data Availability

Data supporting the conclusions of this article will be made available by the corresponding author, Zhiwei Wu, upon reasonable request, considering the restricted nature of the data. All data are stored securely on Case Yili Normal University’s institutional Box platform, which provides controlled access and long-term preservation in line with university policies. The data are guaranteed to be retained for a period of at least 5 years.
